# Prediction of preoperative peritoneal cancer index for pseudomyxoma peritonei by multiple linear regression analysis

**DOI:** 10.3389/fmolb.2024.1512937

**Published:** 2024-12-23

**Authors:** Mingjian Bai, Jing Feng, Jie Liu, Yunxiang Li, Yueming Xu, Fucun Ma, Ruiqing Ma, Guowei Liang, Xuekai Liu, Na Zhao

**Affiliations:** ^1^ Department of Clinical Laboratory, Aerospace Center Hospital, Beijing, China; ^2^ Department of Literature and Science, University of Wisconsin-Madison, Madison, WI, United States; ^3^ Department of Myxoma, Aerospace Center Hospital, Beijing, China; ^4^ Department of Nephrology, Aerospace Center Hospital, Beijing, China

**Keywords:** pseudomyxoma peritonei, peritoneal cancer index, prediction, multiple linear regression, surgery

## Abstract

**Background:**

The aim of the present study was to establish a predictive model to predict the peritoneal cancer index (PCI) preoperatively in patients with pseudomyxoma peritonei (PMP).

**Methods:**

A total of 372 PMP patients were consecutively included from a prospective follow-up database between 1 June 2013 and 1 June 2023. Nine potential variables, namely, gender, age, Barthel Index (BAI), hemoglobin (Hb), albumin (Alb), D-dimer, carcinoembryonic antigen (CEA), carbohydrate antigen 125 (CA 125), and CA 19-9, were estimated using multiple linear regression (MLR) analysis with a stepwise selection procedure. The established MLR model was internally validated using *K*-fold cross-validation. The agreement between the predicted and surgical PCI was assessed using Bland–Altman plots and intraclass correlation (ICC). A *p-*value of less than 0.05 was considered statistically significant.

**Results:**

Six independent predictors were confirmed by the stepwise MLR analysis with an *R* ^*2*^ value of 0.570. The predicted PCI formula was represented as follows: PCI = 19.567 + 2.091 * Gender (male = 1, female = 0) − 0.643 * Alb +4.201 * Lg (D-dimer) + 2.938 * Lg (CEA) + 5.441 * Lg (CA 125) + 1.802 * Lg (CA 19-9). The agreement between predicted and surgical PCI was assessed using Bland–Altman plots, showing a limit of agreement (LoA) between −15.847 (95%*CI*: −17.2646 to −14.4292) and +15.847 (95%*CI*: 14.4292–17.2646).

**Conclusion:**

This study represents the first attempt to use an MLR model for the preoperative prediction of PCI in PMP patients. Nevertheless, the MLR model did not perform well enough in predicting preoperative PCI. In the future, more advanced statistical techniques and a radiomics-based CT-PCI-participated MLR model will be developed, which may enhance the predictive ability of PCI.

## Background

Pseudomyxoma peritonei (PMP) is a rare clinical entity characterized by diffuse intra-abdominal gelatinous ascites with mucinous implants on the peritoneal surfaces (Yu, 2019), which mainly originates from the appendix (Darr et al., 2017). The estimated annual incidence of PMP is approximately 1–4 cases per million (Mittal et al., 2017). The currently recommended standard treatment strategy for PMP is complete cytoreduction surgery (CRS) combined with hyperthermic intraperitoneal chemotherapy (HIPEC) (Li et al., 2014). In PMP operation, the completeness of cytoreduction (CCR) has been identified as one of the most important prognostic factors for PMP (Mittal et al., 2017), which is consistent with our previous research (Bai et al., 2021).

The peritoneal cancer index (PCI) is used to estimate the tumor burden caused by peritoneal metastases and is negatively correlated with the chances of cytoreduction (Flicek et al., 2016). In patients with colorectal cancer, Sugarbaker et al. first identified a PCI score of at least 20 as commonly representing unresectable disease, while a score less than 20 indicates potentially resectable disease (Da and Sugarbaker, 2006). For PMP, several studies have confirmed that the optimal cut point for PCI in predicting surgical resectability is higher than 20 and varies depending on the population included in the study (Flicek et al., 2016; Votanopoulos et al., 2018; Young et al., 2020). Although PCI could contribute to preoperative patient selection and/or information, the most accurate PCI could only be acquired through laparoscopy or laparotomy (defined as surgical PCI).

In our previous study, we evaluated the correlation between routine laboratory test markers and PCI; however, Spearman’s correlation values between D-dimer, carcinoembryonic antigen (CEA), carbohydrate antigen 125 (CA 125), CA 19-9, and PCI were only 0.487, 0.509, 0.469, and 0.499, respectively (Feng et al., 2022). A former research concluded that computed tomography (CT)-calculated PCI (CT-PCI) could assess tumor burden preoperatively and select patients for whom complete resection was achievable (Bouquot et al., 2018). However, the agreement between surgical PCI and CT-PCI is not ideal (*R* = 0.64) (Flicek et al., 2016). In our recent research, we performed a Bland–Altman agreement analysis between the CT-predicted and surgical PCI in PMP of appendiceal origin, with the limit of agreement ranging from −5.459 to 6.321. This is because of the difficulty in distinguishing the boundary between tumor tissue and mucus based on CT, especially for the small intestine regions, which caused overestimation or underestimation by CT-PCI. Hence, CT-PCI cannot predict surgical PCI accurately even in professional PMP treatment centers (Bai et al., 2023). Therefore, accurately predicting PCI preoperatively using a single predictor appears to be very challenging.

The present study aimed to predict surgical PCI preoperatively in PMP patients by multiple linear regression (MLR) analysis. Predictor variables consisted of both clinical baseline data and biomarkers. First, all potential predictor factors related to PCI were preliminarily screened. Then, all the relevant predictors were included in the MLR model and further underwent internal validation.

## Methods

### Patients

**FIGURE 1:**
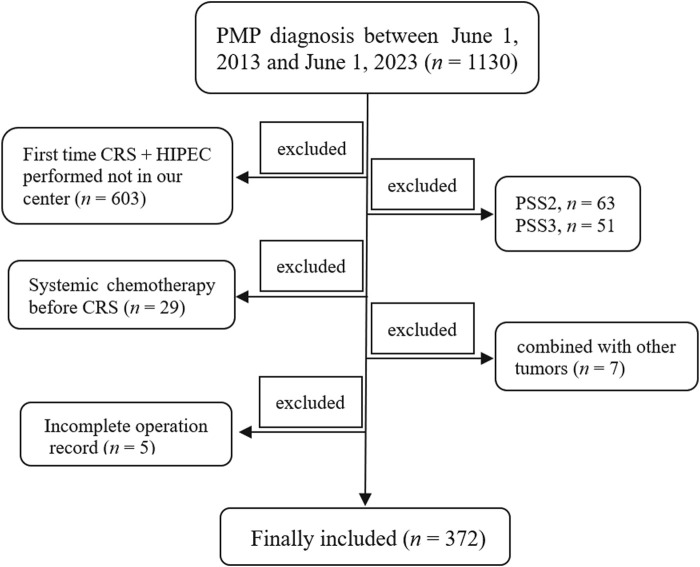
Schematic representation of the study . A total of 1,130 patients with PMP diagnosis were retrieved between 1 June 2013 and 1 June 2023. Patients whose first time CRS + HIPEC was not performed at our center (*n* = 603), whose PSS was not less than 2 (PSS 2, *n* = 63; PSS 3, *n* = 51), who received systemic chemotherapy before CRS (*n* = 29), who were combined with other tumors (*n* = 7), and who had an incomplete surgical record (*n* = 5) were all excluded. Finally, 372 patients were included in the present study. PMP, pseudomyxoma peritonei; CRS, cytoreductive surgery; HIPEC, hyperthermic intraperitoneal chemotherapy; PSS, prior surgical score.

The Institutional Review Board (IRB) of the Aerospace Center Hospital approved the present study (No. 2022-002), and all patients signed an informed consent before undergoing the operation. A total of 1,130 patients diagnosed with PMP were retrieved from the follow-up database of the Myxoma Department between 1 June 2013 and 1 June 2023. The inclusion criterion is as follows: patients who underwent their first standard CRS + HIPEC procedure at our center. The exclusion criteria were as follows: (a) the patient’s first standard CRS + HIPEC procedure was not performed in our center (*n* = 603); (b) prior surgical score (PSS) not less than 2 (PSS 2, *n* = 63; PSS 3, *n* = 51); (c) received systemic chemotherapy before CRS (*n* = 29); (d) patients with concomitant other tumors (one with nasopharyngeal carcinoma, one with lymphoma, one with oral cancer, one with lung cancer, one with breast cancer, one with gastric cancer, and one suffered with both breast and thyroid cancer, *n* = 7); and (e) incomplete surgical record (*n* = 5). Ultimately, 372 subjects were included in the present study (Figure 1). All procedures were performed by the same surgical team.

### Predictor variables

The principle for selecting variables is that they are easy to obtain in clinical work and can be measured objectively and accurately, including both basic clinicopathologic information and biomarkers.

Clinicopathologic variables include gender, age, and the Barthel Index (BAI). The BAI is a widely used scale for measuring the activities of daily living (ADL). There are 10 items in the BAI, ranging from 0 (complete dependence on ADL) to 100 points (complete independence on ADL) (Zhou et al., 2021). The BAI assessment was completed by an oncology-trained specialty nursing team.

Routine laboratory test indicators include hemoglobin (HB), albumin (ALB), and D-dimer. Anemia is a well-documented phenomenon that occurs in patients with cancer (Farag et al., 2023), while ALB is recognized as a nutritional status parameter and is associated with chronic inflammation (McMillan et al., 2001). In addition, our previous research confirmed the correlation between D-dimer and PCI in PMP patients (Feng et al., 2022). Based on the conclusions of the previous studies, we consider that the three traditional markers are likely to have a good correlation with PCI; therefore, the three indicators were all included. The three tumor markers commonly used in PMP are CEA, CA 125, and CA 19-9 (Taflampas et al., 2014). The detection method for the three tumor markers was detailed in our previously published work (Bai et al., 2022).

### Surgical PCI calculation

PCI represents the most accurate system to draw a detailed preoperative map of carcinomatosis of PMP for the surgeon. Surgical PCI calculation refers to the method described by Sugarbaker and Jablonski (1995).

### Statistical analysis

All statistical analyses were performed using SPSS (version 16.0; IBM Corporation), MedCalc (version 15.2.2; MedCalc Software, Flanders, Belgium), GraphPad prism (version 8.0; GraphPad Software), and R (version 4.0.5; R Foundation for Statistical Computing, Vienna). All continuous data between groups were compared using the *t*-test or Mann–Whitney U test, as appropriate.

The “*pwr*” package in *R* was used for sample size calculations. The preset medium effect size was *f* = 0.15 (Darr et al., 2017), the statistical power (1- *β*) was 0.80, and the significance level (*α*) was 0.05. Subsequently, it was calculated that at least 113 PMP patients were required for the MLR model to be constructed in this study. The statistical process consists of three consecutive steps, including screening for predictor variables, building the MLR model, and verifying the model.

First, the preliminary screening of predictive variables was performed before the MLR analysis. The initial judgment of a possible relationship between the predictive continuous variables and PCI was made according to the scatter plot, which shows whether the relationship is linear or non-linear preliminarily (Schneider et al., 2010). D-dimer and the three tumor markers all underwent logarithmic transformation (*f* (x) = Lg (x)) in order to meet the conditions for linear regression application (Yu et al., 2020). For continuous variables, only the correlation between predictors and PCI not less than 0.30 was included for further MLR analysis in order to avoid overfitting of the model. Gender, as a binary variable, was also included because it has been demonstrated to have different PCI levels in PMP patients (Feng et al., 2022).

Second, a stepwise selection procedure was performed during the MLR analysis. The initially established regression model was further checked for residual independence (Durbin–Watson test), normality, and homogeneity of variance (Levene test). The multicollinearity between predictor variables was assessed using the variance inflation factor (VIF) (Banerjee et al., 2019). The *R* (multiple regression coefficient) and adjusted *R* ^2^ values were also calculated. Multiple regression coefficient values >0.7 were regarded as a “strong” correlation, values between 0.50 and 0.70 were interpreted as a “good” correlation, values between 0.3 and 0.5 were treated as a “fair” or “moderate” correlation, and any value < 0.30 was considered a poor correlation. The *R* ^2^ statistic provided information about the goodness-of-fit of a model, with an *R* ^2^ value of 1, indicating that the regression line perfectly fit the data (Hazra and Gogtay, 2016).

Finally, the model estimation was completed using the widely used *K*-fold cross-validation (CV) methods (Jung and Hu, 2015). In a typical *K*-fold CV procedure for a linear model, the dataset is randomly and evenly split into *K* parts (if possible). A candidate model is built based on *K* - 1 parts of the dataset, called a training set. The predictive accuracy of this candidate model is then evaluated on a test set containing the data from the hold-out part. This model validation process was performed using the *R* project, which calculated the root mean square error (*RMSE*) and mean absolute error (*MAE*) to internally validate our established model. Subsequently, based on the Bland–Altman plot and intraclass correlation (*ICC*), the agreement assessment between the predicted PCI and surgical PCI was performed (Bland and Altman, 1986). The *ICC* coefficient boundaries <0.4 were regarded as poor; 0.40–0.60 as fair; 0.60–0.74 as good; and >0.75 as strong (Hazra and Gogtay, 2016). A *p-*value less than 0.05 was considered statistically significant.

## Results

The median (interquartile range, IQR) PCI of the included 372 patients (208 male patients and 164 female patients) was 27 (14, 32). The mean (standard deviation, SD) age was 57.0 ± 11.4 years. The median BAI was 100 (95, 100). According to the Peritoneal Surface Oncology Group International (PSOGI) criteria, the histopathological grading includes four subtypes: acellular mucin (*n* = 1), disseminated peritoneal adenomucinosis (DPAM) (*n* = 276), peritoneal mucinous carcinomatosis (PMCA) (*n* = 67), and peritoneal mucinous carcinomatosis with signet ring cells (PMCA‐S) (*n* = 28). The detailed clinicopathologic characteristics are shown in Table 1.

**TABLE 1:** Clinicopathologic features of 372 included patients with PMP.

Characteristic	NO./level
Gender (male/female)	208/164
Age (years)	57.0 ± 11.4
Hospital time (days)	24 (20, 27)
Barthel Index score	100 (95, 100)
Missing data (*n* = 2)	
Albumin (g/L)	37.0 ± 5.0
Missing data (*n* = 1)	
Hemoglobin (g/L)	123 ± 18
Lg (D-dimer) (ng/mL)	2.56 ± 0.54
Missing data (*n* = 2)	
Lg (CEA) (ng/mL)	1.12 (0.38, 1.75)
Lg (CA 125) (U/mL)	1.68 (1.21, 2.04)
Lg (CA 19-9) (U/mL)	1.39 (0.86, 2.20)
PCI	27 (14, 32)
Degree of radical surgery	
CCR 0/1 (*n*)	152
CCR 2/3 (*n*)	220
Pathology	
Acellular mucin (*n*)	1
DPAM (*n*)	276
PMCA (*n*)	67
PMCA-S (*n*)	28

PMP, pseudomyxoma peritonei; PCI, peritoneal carcinomatosis index; CCR, completeness of cytoreduction; DPAM, disseminated peritoneal adenomucinosis; PMCA, peritoneal mucinous carcinomatosis; PMCA-S, peritoneal mucinous carcinomatosis with signet ring cells.

**FIGURE 2:**
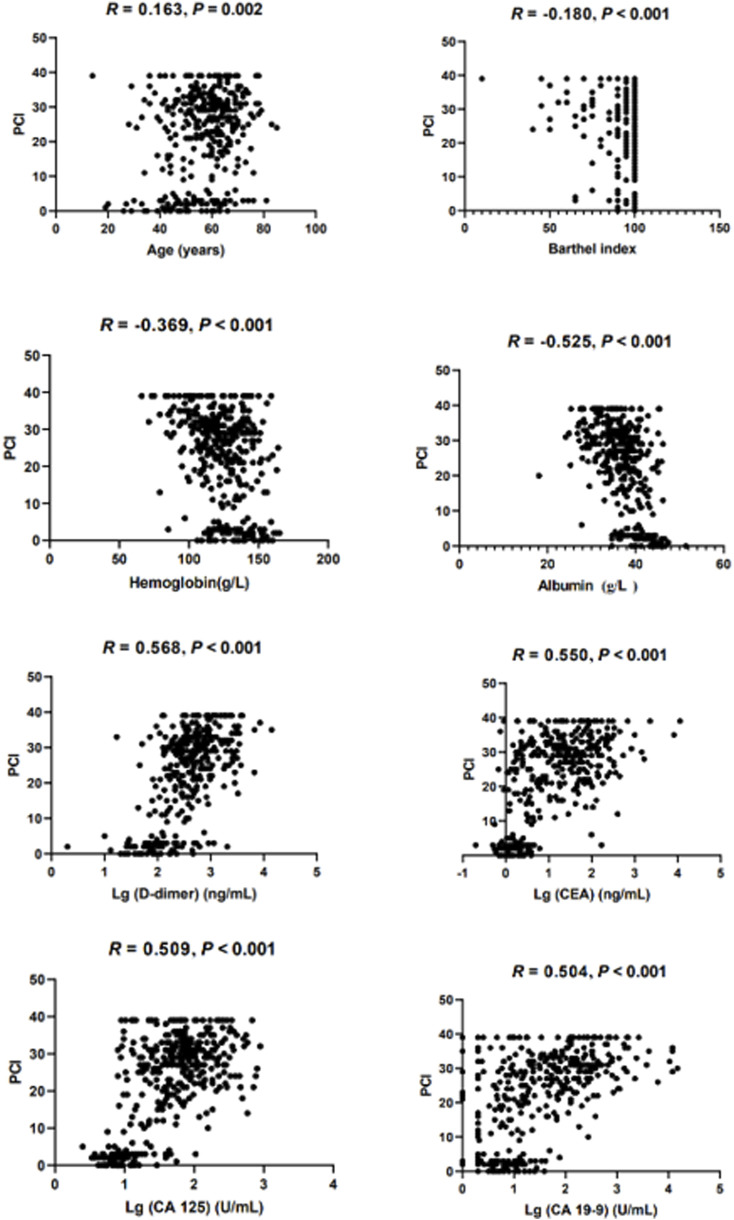
Scatterplot between all continuous predictor variables and PCI.

The median PCI between male patients and female patients was 29 (16, 34) *vs.* 24 (11, 30), *Z* = −3.369, and *P* < 0.001. The intraoperative PCI did not conform to a normal distribution (kurtosis = −0.972, skewness = −0.591). Followed, Spearman’s rank correlation was used to analyze the correlation between the predictor variables and PCI. The correlation coefficients between the PCI and age, BAI, Hb, Alb, Lg (D-dimer), Lg (CEA), Lg (CA 125), and Lg (CA 19-9) were 0.163 (*P* = 0.002), −0.180 (*P* < 0.001), −0.369 (*P* < 0.001), −0.525 (*P* < 0.001), 0.568 (*P* < 0.001), 0.550 (*P* < 0.001), 0.509 (*P* < 0.001), and 0.504 (*P* < 0.001), respectively (Figure 2). Finally, seven predictor variables (gender, Hb, Alb, Lg (D-dimer), Lg (CEA), Lg (CA 125), and Lg (CA 19-9)) were included in the MLR analysis.

In the MLR analysis using a stepwise method, the *F* value of 81.932 (*P* < 0.001) was obtained. The Durbin–Watson statistic was 1.070, indicating that the residuals were independent. The histogram of the standard regression residuals accorded with a normal distribution (Supplementary Figure S1). The standard residuals fluctuated around the zero-scale line, suggesting homogeneity of variance (Supplementary Figure S2). The VIF showed that there was no collinearity problem between the predictor variables. Finally, the present study confirmed six independent predictors associated with PCI: gender, Alb, D-dimer, CEA, CA 125, and CA 19-9. The predicted PCI formula is as follows: PCI = 19.567 + 2.091 * Gender (male = 1, female = 0) − 0.643 * Alb + 4.201 * Lg (D-dimer) + 2.938 * Lg (CEA) + 5.441 * Lg (CA125) + 1.802 * Lg (CA 19-9). The details are shown in Table 2.

**TABLE 2:** Multiple linear regression analysis for predicting PCI in patients with PMP.

	Unstandardized coefficient		Standardized coefficient	*t*	*P*	Collinearity statistics	
	*B*	Standard error	*Beta*			Tolerance	VIF
Constant	19.567	5.176		3.781	0.001		
Gender	2.091	0.863	0.084	2.422	0.016	0.983	1.017
Alb	−0.643	0.100	−0.257	−6.457	0.001	0.739	1.353
Lg (D-dimer)	4.201	1.119	0.181	3.752	0.001	0.502	1.993
Lg (CEA)	2.938	0.658	0.197	4.463	0.001	0.601	1.663
Lg (CA 125)	5.441	1.040	0.239	5.231	0.001	0.560	1.787
Lg (CA 19-9)	1.802	0.584	0.129	3.083	0.002	0.670	1.493

PCI, peritoneal cancer index; PMP, pseudomyxoma peritonei; VIF, variance inflation factor. Gender (male = 1, female = 0).

**FIGURE 3:**
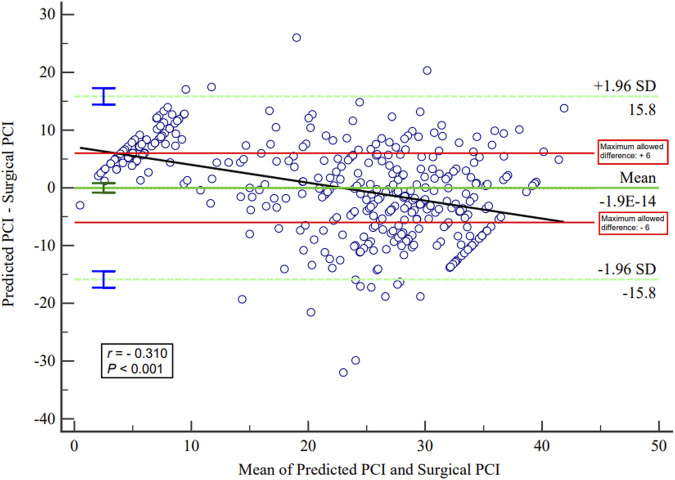
Bland–Altman plots of surgical and predicted PCI.

**FIGURE 4:**
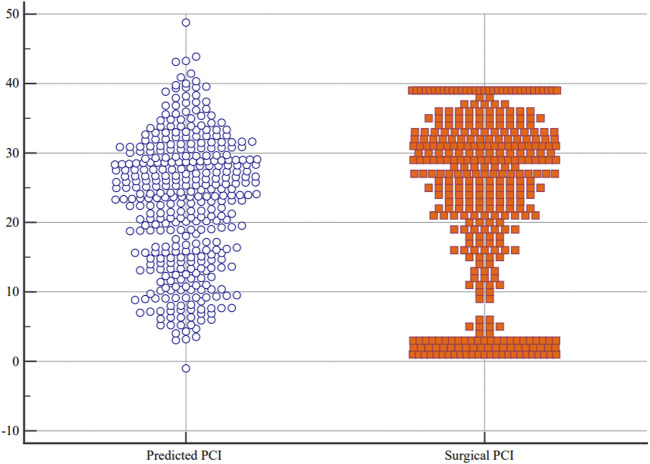
Distribution of predicted and surgical PCI by the multiple dot plots.

The present study used 10-fold cross-validation for estimating the established MLR model. The *R* ^*2*^ value was 0.570, MAE was 6.599, and RMSE was 8.110. The Bland–Altman plots between the surgical PCI and predicted PCI using the present equation are described in Figure 3. The plots indicated a significant but weak correlation between the mean predicted and surgical PCI and the difference between the predicted and surgical PCI (*r* = −0.310). The limit of agreement (LoA) ranged from −15.847 (95% *CI*: −17.2646 to −14.4292) to +15.847 (95% *CI*: 14.4292–17.2646). In clinical practice, a discrepancy of ±5–7 PCI points may not be clinically meaningful if it does not influence the ultimate outcome of the surgery (Flicek et al., 2016). Based on this, the maximum allowed difference between methods was defined as 6. The ICC between the surgical and predicted PCI was 0.731 (95% *CI*: 0.680–0.776), and the multiple dot plots (plotting all data) are shown in Figure 4.

## Discussion

The present study developed an MLR model for predicting preoperative PCI with an *R* ^*2*^ value of 0.570. Subsequently, the MLR model underwent 10-fold cross-validation, resulting in an RMSE value of 8.110. To the best of our knowledge, this is the first MLR model to predict PCI preoperatively in PMP patients.

Male PMP patients appeared to have significantly higher PCI levels than female patients. In the MLR analysis, the gender factor was an independent predictor of preoperative PCI assessment. Two reasons, perhaps, could explain this phenomenon. First, female patients often present with rapidly enlarging ovarian masses and consequently tend to become symptomatic, or they are clinically obvious, whereas male patients are often asymptomatic initially (Smeenk et al., 2008; Jarvinen and Lepisto, 2010). In short, male patients tend to present at a more advanced stage than female patients. Additionally, earlier presentation in female subjects may also result from a more liberal use of cross-sectional imaging in women with suspected ovarian cancer (Mittal et al., 2017). Therefore, male PMP patients often have a higher tumor burden than female patients before the operation.

The present research found that Alb was the independent predictor with the largest standardized coefficient in the PCI predictive equation. Although PMP is often asymptomatic in the initial stages, it gradually presents with vague abdominal symptoms (such as abdominal distension, discomfort, and pain, and with palpable abdominal masses). When the disease burden is marked, PMP patients eventually experience malnutrition, bowel obstruction, and other complications (Mittal et al., 2017). Alb has been advocated as a useful marker for assessing nutritional status (Thomas et al., 2000), which has even been proven to have an additive effect on mortality (Corona et al., 2018). Hence, when PMP patients experience malnutrition, especially in tumor cachexia, the Alb levels often decrease significantly. Additionally, a previous study confirmed that the Alb-participated modified Glasgow prognostic score (mGPS) could be used as a cost-effective prognostic tool for predicting overall survival (OS) and disease-free survival (DFS) in PMP patients (Tan et al., 2017). To the best of our knowledge, there are still few studies on the application value of albumin in PMP patients. In the future, more research studies are needed to confirm the above conclusions, especially the need for multi-center large sample studies.

Tumor markers have been widely assessed in PMP patients; of these, the most commonly determined markers were CEA, CA 125, and CA 19-9. According to literature reviews and our previous research results, CA 19-9 may be the most valuable tumor marker for PMP, which not only acts as an independent prognostic indicator for PMP (Bai et al., 2021; Koh et al., 2013) but could also help predict the completeness of cytoreduction (Bai et al., 2022). The present study even found that CA 19-9 can independently predict the tumor burden of PMP. CA125 is another useful tumor marker in PMP, as it can be elevated in patients with any source of peritoneal irritation (Mittal et al., 2017). CEA, which is expressed by tumors of the gastrointestinal tract, particularly colorectal cancer, may also reflect the tumor burden in PMP patients.

One of the most common complications associated with cancer is the development of a coagulation disorder (Horowitz et al., 2011), and irregularities in coagulation and fibrinolysis are often observed in cancer patients (Lee, 2002). Our previous research confirmed that D-dimer levels were positively correlated with PCI in PMP patients (Feng et al., 2022). In the present research, D-dimer could serve as an independent predictor in the MLR model to predict PCI preoperatively in PMP patients. Previous research even found that D-dimer levels were independently associated with OS in PMP patients (Bai et al., 2021). Therefore, several studies have confirmed that D-dimer is a very useful biomarker for PMP patients that can be integrated into prognostic models. We believe that there may be two reasons for the increase of D-dimer levels in PMP patients. First, as a solid tumor, PMP itself may cause an increase in D-dimer. Second, in clinical practice, we found that PMP patients were prone to deep venous thrombosis (DVT), which was also reported with a rate of 15.7% (11/70) in a previous study (Shiralkar et al., 2017). With the increase in tumor burden, the intra-abdominal pressure of PMP patients also increases, leading to lower extremity venous reflux disorder. This makes it easier for a thrombus to form, resulting in increased D-dimer levels.

Although the predictor variables included in the MLR model are routinely detected indicators and can be easily acquired in clinical practice, the developed model in the present study still seems to be unable to meet the clinical needs after verification. The RMSE was 8.110 in the present MLR model, indicating that the model still needs further improvement to enhance its predictive ability. In the Bland–Altman analysis, the 95% CI of the LoAs exceeded the clinically acceptable range, suggesting that the currently established model cannot accurately predict PCI before the operation. The ICC between the surgical and predicted PCI was not good enough in the present research. Upon careful observation of the multiple dot plots of predicted and surgical PCI, it can be seen that the predictive ability of the model is poor when PCI is less than 10 or greater than 35. Therefore, it is necessary to improve the predictive ability of PCI in this range.

There were several limitations to the present study. First, the predictive variables included in the *MLR* model are still insufficient. For instance, abdominopelvic CT is considered to be the best imaging modality for the detection of peritoneal tumors (Qi et al., 2017). Nevertheless, only 167 out of 372 patients received preoperative CT-PCI in our research. In order to ensure the number of cases and avoid selection bias, CT-PCI was not included in the MLR analysis temporarily. Second, the RMSE, Bland–Altman, and ICC analyses all indicated the limited predictive ability of the model established in this study, which still cannot accurately predict PCI before surgery. Third, the present study only underwent internal validation but not external validation, which limits the generalizability ability of the model. In addition, the predictive power of the model needs further refinement and improvement in the future.

## Conclusion

To conclude, the preoperative prediction of PCI not only contributes to better preoperative patient selection but also provides more informed consent to patients. To the best of our knowledge, the present study innovatively attempted to use the MLR model to predict PCI levels for PMP patients. Nevertheless, the developed MLR model did not perform well enough in predicting preoperative PCI. In the future, more advanced statistical techniques and the radiomics-based CT-PCI-participated MLR model may improve the predictive ability of PCI for PMP patients.

## Data Availability

The raw data supporting the conclusions of this article will be made available by the authors, without undue reservation. Requests to access the datasets should be directed to liangguowei721@126.com.

## Supplementary material

SUPPLEMENTARY FIGURE 1 Histogram of the standard regression residual in multiple linear regression analysis.

SUPPLEMENTARY FIGURE 2 Scatter between the standardized predicted value and standardized residual in multiple linear regression analysis.

## Abbreviations

PMP, pseudomyxoma peritonei; CRS, complete cytoreduction surgery; HIPEC, hyperthermic intraperitoneal chemotherapy; PCI, peritoneal cancer index; MLR, multiple linear regression; BAI, Barthel Index; ADL, activity of daily living; Hb, hemoglobin; Alb, albumin; VIF, variance inflation factor; CV, cross-validation; RMSE, root mean square error; MAE, mean absolute error; ICC, intraclass correlation coefficient; DPAM, disseminated peritoneal adenomucinosis; PMCA, peritoneal mucinous carcinomatosis; PMCA‐S, peritoneal mucinous carcinomatosis with signet ring cells; mGPS, modified Glasgow prognostic score; DVT, deep venous thrombosis.
