# Supplementary figures and images for: Prediction of preoperative peritoneal cancer index for pseudomyxoma peritonei by multiple linear regression analysis
Source: Front Mol Biosci. 2024 Dec 23;11:1512937. doi: 10.3389/fmolb.2024.1512937 (PMC11700817; doi:10.3389/fmolb.2024.1512937)

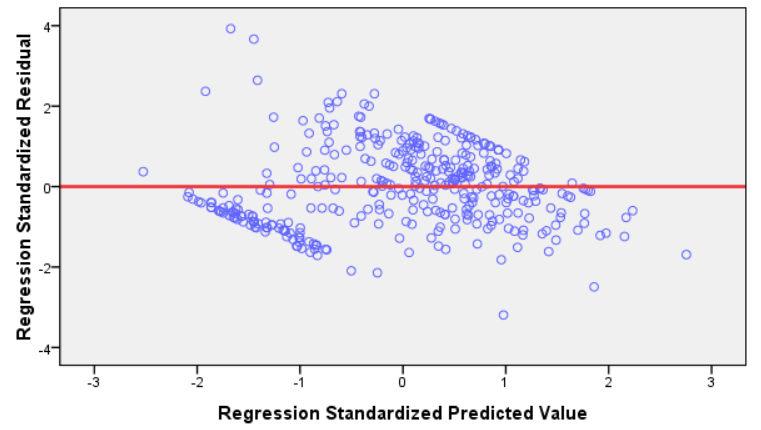

Supplement: Supplementary file 1 [file Image2.tif]

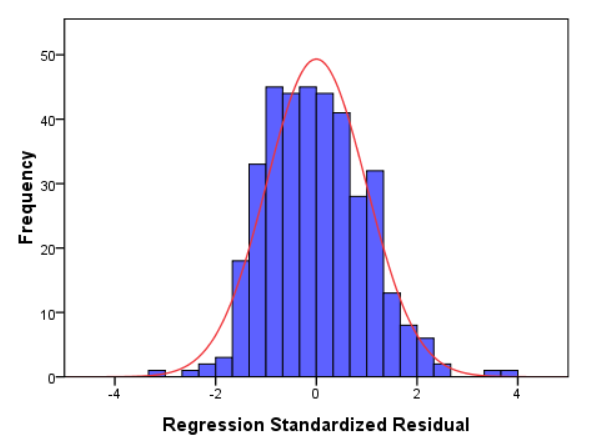

Supplement: Supplementary file 2 [file Image1.tif]
